# Reliability of end, stable, neutral, first coronal reverse vertebrae identification in degenerative lumbar scoliosis: Intra- and interobserver consistency analysis

**DOI:** 10.3389/fsurg.2023.1116590

**Published:** 2023-02-13

**Authors:** Hui Wang, Xiao Liang, Jiaxin Xu, Jiayuan Sun, Dalong Yang, Weishi Li, Wenyuan Ding

**Affiliations:** ^1^ Spine Department, Hebei Medical University Third Hospital, Shijiazhuang, China; ^2^ Department of Orthopaedic, Peking University Third Hospital, Beijing, China

**Keywords:** degenerative lumbar scoliosis, first coronal reverse vertebrae, intraobserver reliability, coronal malalignment, observer training level

## Abstract

**Objective:**

To assess the intra- and interobserver reliability by observer training level used for selecting the end vertebra (EV), neutral vertebra (NV), stable vertebra (SV), and first coronal reverse vertebrae (FCRV) in degenerative lumbar scoliosis (DLS) patients.

**Methods:**

Fifty consecutive upright long-cassette radiographs and CT examination of operative cases of DLS were evaluated by three surgeons at various levels of training. For each iteration, the observers attempted to identify the UEV, NV and SV from x-ray, and FCRV from the CT examination. Intra- and interobserver reliability was assessed by means of Cohen's Kappa correlation coefficient, and raw percentages of agreement were recorded.

**Results:**

Intraobserver reliability was excellent for determining FCRV (*K*_a_ = 0.761–0.837), fair to good for determining UEV (*K*_a_ = 0.530–0.636), fair to good for determining SV (*K*_a_ = 0.519–0.644), and fair to good for determining NV (*K*_a_ = 0.504–0.734), respectively. Additionally, we also noted a trend towards better intraobserver reliability with increasing levels of experience. Interobserver reliability was poor between observers beyond chance for UEV, NV, SV (*K*_a_ = 0.105–0.358), and good reliability for FCRV (*K*_a_ = 0.581–0.624). All three observers agreed on the same level of the FCRV in 24 patients of the time, which presented less Coronal imbalance type C compared to the other 26 patients.

**Conclusion:**

Experience and training level of the observers are important factors affecting the accurate identification of these vertebrae in DLS, intraobserver reliability increases along with increasing levels of observer experience. FCRV is superior to UEV, NV, and SV in the accuracy of identification, Type C coronal malalignment could affect the accurate identification of FCRV.

## Introduction

Selection of upper instrumented vertebrae (UIV) has been proved to be closely related to the postoperative proximal adjacent segment degeneration and proximal scoliosis progression followed posterior lumbar fusion for degenerative lumbar scoliosis (DLS), which are common radiological findings but may progressed to adjacent segment disease that required revision surgery (1–4). Bridwell et al. (5) stated that choosing proximal fusion level requires identification of the stable vertebra (SV), neutral vertebra (NV), upper end vertebrae (UEV) from the x-ray examination. This process is a prerequisite for achieving both maximal curve correction and a stable, well-balanced spine while fusing as few motion segments as possible (6). For determination of the optimal proximal fusion level, Wang et al. defined a new concept named first coronal reverse vertebrae (FCRV) based on Hounsfield unit (HU) measurement from computed tomography (CT) examination, which is the first vertebrae that presents opposite orientation of asymmetric HU ratio from the other vertebrae within the major curve (7, 8). Proximal fusion level above FCRV could decrease the risk of postoperative proximal scoliosis progression in DLS when compared to the SV.

FCRV represents the transitional point of the mechanical load and may be within a more stable condition than SV measured from radiographs, the reliability and accuracy of vertebra HU measurement are not affected by the posture, it is reasonable to believe that FCRV is more reliable and objective than SV in the preoperative evaluation of UIV for DLS patients (7, 8). However, no previous study has attempted to assess the reliability and reproducibility in determining the FCRV, SV, NV, UEV in DLS patients, little study specifically focus on the superiority of FCRV in the identification and interpretation. The purpose of the present investigation was to assess the intra- and interobserver reliability of selecting the SV, NV, UEV, and FCRV among three surgeons with varying levels of training based on standing posterior anterior preoperative x-ray examination and CT examination.

## Materials and methods

### Study design and patients

This retrospective study was approved by the Institutional Review Board of the Third Hospital of HeBei Medical University (H2022206056). Before data collection and analysis, each patient provided informed consent.

Inclusion criteria: (1) DLS patients with age older than 50 years. (2) Full-spine Postero-Anterior (P/A) x-ray. (3) Lumbar CT was available for HU measurement. Exclusion criteria: (1) Previous surgery for degenerative lumbar disease. (2) Spinal infections or metabolic disease that may potentially affect accuracy of HU measurements. (3) The anatomical identification was difficult to recognize for radiological measurement.

Fifty consecutive upright long-cassette radiographs and CT examination of operative cases of DLS were evaluated on the same occasion by three surgeons at various levels of training [fellowship-trained spine surgeon (observer 1; JX), fellow in-training (observer 2; JS), orthopaedic surgery resident (observer 3; XL)]. The radiograph order was scrambled between each measurement iteration by each observer. For each iteration, the observers attempted to identify the upper end vertebrae (UEV), neutral vertebrae (NV) and stable vertebrae (SV) from x-ray, and first coronal reverse vertebrae (FCRV) from the CT examination.

**Figure 1:**
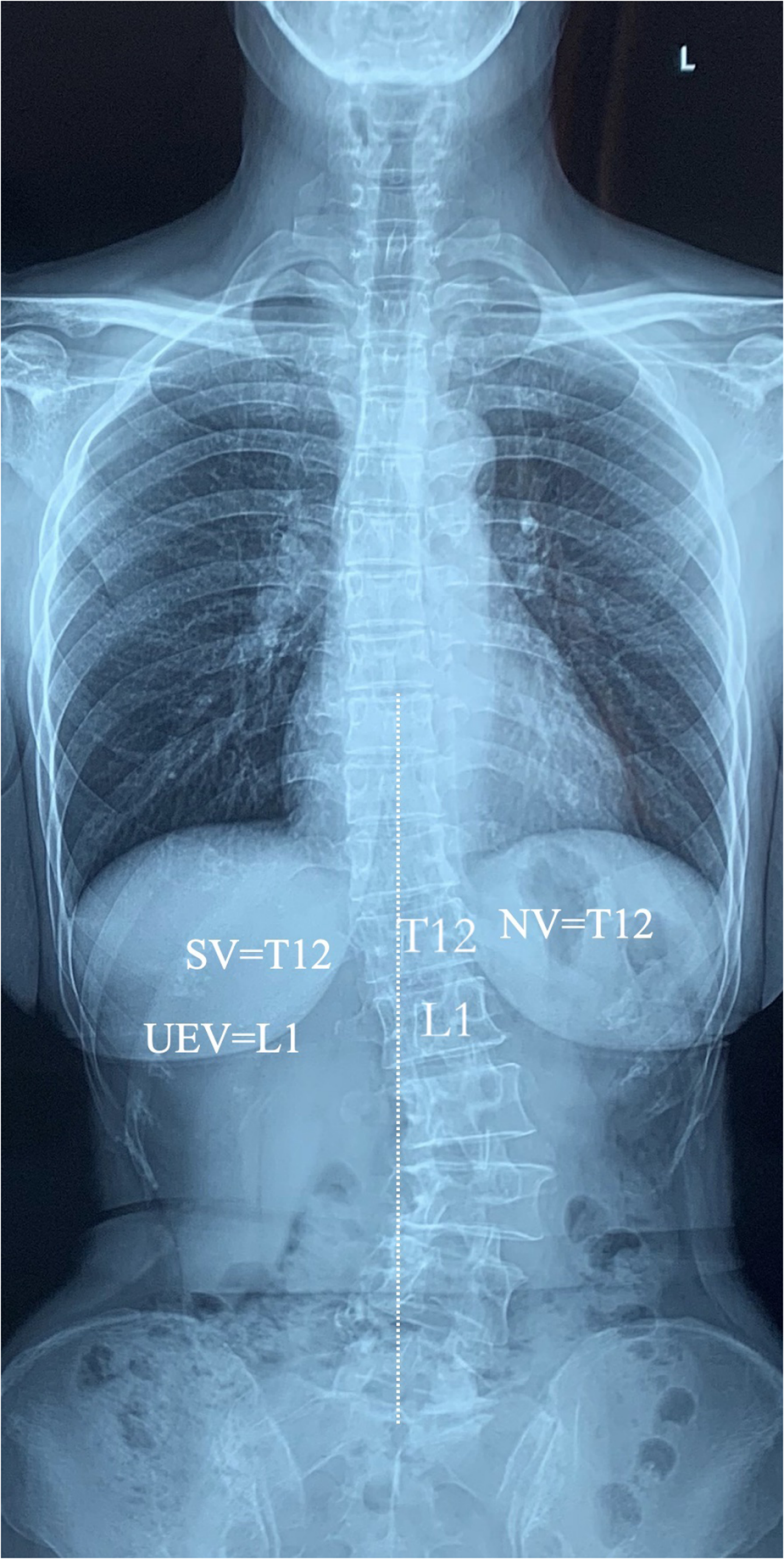
Schematic demonstrating the upper end vertebra, neutral vertebra (not rotated; pedicles and body symmetric, spinous process midline), and stable vertebra (CSVL bisects pedicles).

**Figure 2:**
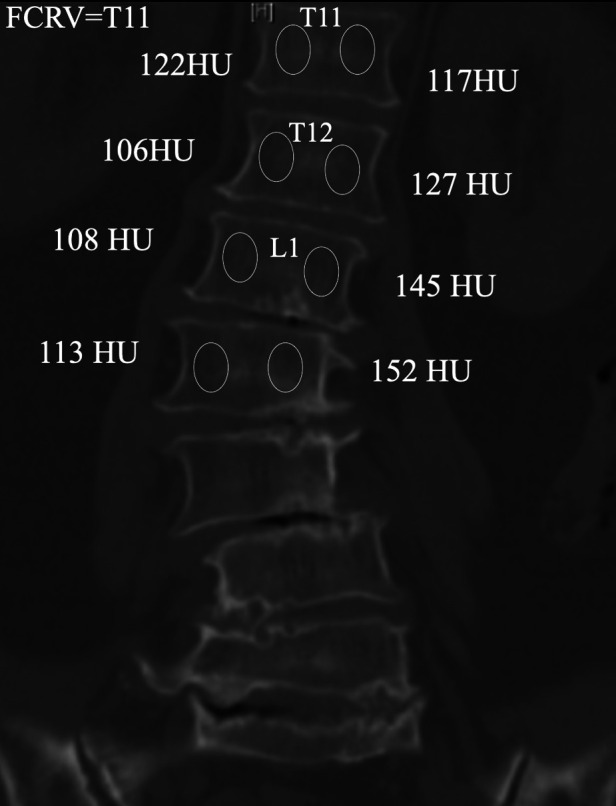
Schematic diagram of FCRV measurement, which is defined as the first vertebrae that presents opposite orientation of asymmetric hounsfield unit (HU) ratio from the other vertebrae within major curve.

The UEV is defined as the most tilted vertebrae (that which subtended the greatest Cobb angle) at the cephalad end of the main curve. The NV is defined as the most cephalad vertebrae with apparently neutral rotation as assessed by pedicle symmetry within the radiographic silhouette of the corresponding vertebrae. The SV is the most cephalad vertebra closest to the end vertebra of the main curve that is most nearly bisected by the central sacral vertical line (CSVL) (Figure 1). When two adjacent levels were felt to equally satisfy the above criteria (e.g., the CSVL perfectly bisected the disc between, but not the bodies of, two adjacent vertebrae), the observers were instructed to select the more proximal level. The FCRV is defined as the first vertebrae that presents opposite orientation of asymmetric Hounsfield unit (HU) ratio from the other vertebrae within major curve (Figure 2).

**Figure 3:**
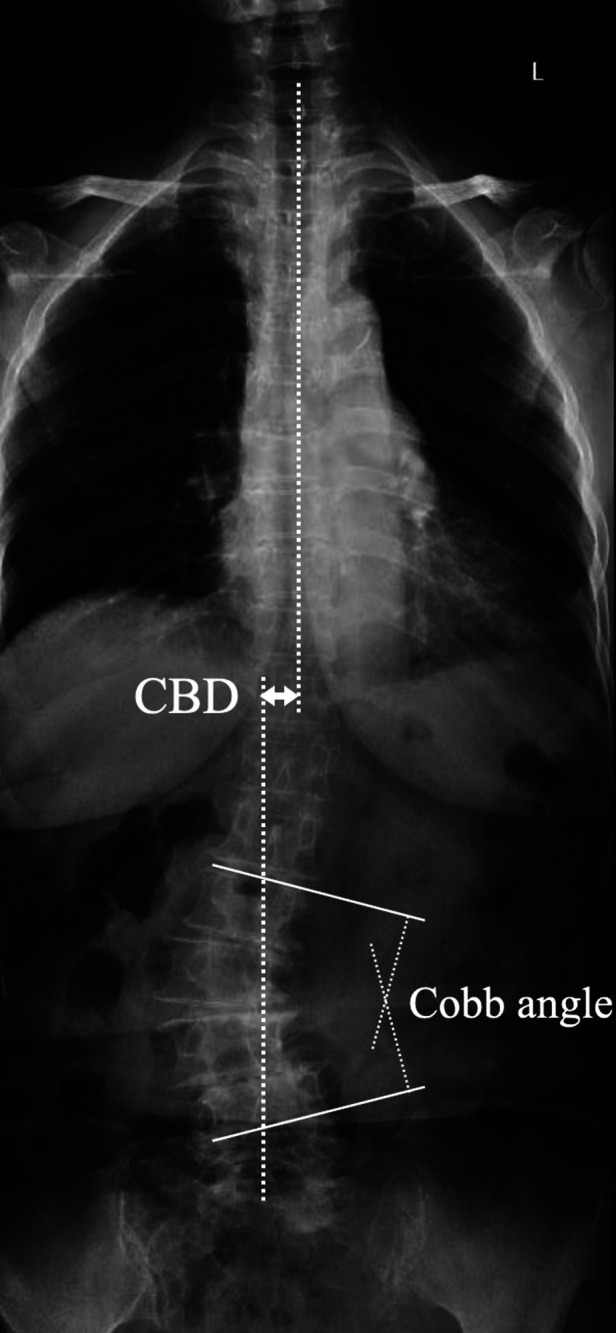
Schematic diagram of coronal spinal parameters measurement. Cobb's angle was measured between the most tilted vertebrae. CBD was the distance between C7 plumb line and CSVL.

All the patients were divided into two groups according to the agreement for FCRV. Age, gender, and bone mineral density (BMD) were recorded. Cobb's angle is measured between the most tilted vertebrae. Coronal balance distance (CBD) is the distance between C7 plumb line and CSVL. Coronal malalignment are classified based on the CBD: Type A, CBD < 3 cm; Type B, CBD > 3 cm and C7PL shifted to the concave side of the curve; Type C, CBD > 3 cm and C7PL shifted to the convex side (Figure 3). All radiographic parameters were measured by two independent observers (first and second author), and were averaged to give a mean value for statistical analysis.

### Statistical analysis

Data were analyzed using Statistical Product and Service Solutions software (version 17; SPSS, Chicago, IL). Three-way Cohen's Kappa correlation coefficients were calculated to assess the intra- and interobserver reliability for determining the UEV, NV, SV and FCRV, respectively. We also adopted *K*_a_ value of 0.75 and above to represent excellent agreement, 0.55–0.74 good agreement, 0.40–0.54 fair agreement, and 0.39 and below poor agreement beyond chance. Continuous variables were recorded as mean ± standard deviation, and categorical variables were expressed as frequency or percentages. An independent *t* test was used to analyze the difference of continuous variables. An *χ*^2^ analysis and Fisher's exact test were used to examine the differences among categorical variables. The statistical significance was set at *p* < 0.05.

## Results

### Characteristics of the subjects

Among the 50 patients included in the current study, 12 males and 38 females, with mean age of 61.7 ± 8.3 years. Coronal imbalance type A was detected in 31 patients, type B was detected in 9 patients, type C was detected in 10 patients. 27 patients presented apex orientation toward left and 23 patients presented apex orientation toward right. The mean Cobb angle was 23.6 ± 5.6 degrees.

### Intraobserver reliability

The *K*_a_ values were excellent for determining the FCRV (*K*_a_ = 0.761–0.837), fair to good for determining the UEV (*K*_a_ = 0.530–0.636), fair to good for determining the SV (*K*_a_ = 0.519–0.644), and fair to good for determining the NV (*K*_a_ = 0.504–0.734). Additionally, we also noted a trend towards better intraobserver reliability with increasing levels of experience; observer 1 demonstrated average *K*_a_ value of 0.713 versus 0.671 for observer 2, and 0.579 for observer 3 (Table 1).

**Table 1:** Intraobserver reliability of SV, NV, UEV, FCRV identification among three surgeons.

	UEV	SV	NV	FCRV
Observer 1	0.636	0.644	0.734	0.837
Observer 2	0.575	0.641	0.684	0.784
Observer 3	0.530	0.519	0.504	0.761

### Interobserver reliability

For the first measurement by each observer, the *K*_a_ values demonstrated poor reliability for agreement between observers beyond chance for UEV, NV, SV (*K*_a_ = 0.105–0.358), and good reliability for FCRV (*K*_a_ = 0.581–0.624). All three observers agreed on the exact level of the UEV 14% of the time, the NV 8% of the time, the SV 12% of the time, and the FCRV 48% of the time. Conversely, all three observers disagreed (each selected a different vertebral level) for 6% of EV, 12% of NV, 10% of SV, and 2% of FCRV.

### Potential factors affecting the reliability in determining FCRV

For the first measurement by each observer, all three observers agreed on the same level of the FCRV in 24 patients of the time, they were enrolled as the High agreement group, the other 26 patients were enrolled as the Low agreement group. There were significant difference in the Coronal imbalance type between the two groups, Coronal imbalance type C was more common in Low agreement group when compared to the High agreement group (Table 2).

**Table 2:** Comparison of clinical and radiological data between high and low agreement groups.

	High agreement group	Low agreement group	Statistics	*p* value
Sex (M/F)	8/16	4/22	2.204	0.138
Age (year)	60.2 ± 7.2	63.1 ± 9.1	−1.251	0.217
Coronal imbalance type				
A	20	11		
B	4	5		
C	0	10	12.664	0.002
Apex orientation				
Left	14	13		
Right	10	13	0.349	0.555
Cobb angle	22.0 ± 3.5	25.0 ± 6.7	−1.921	0.061
L1 HU value	158.4 ± 23.3	146.7 ± 36.8	1.323	0.192

## Discussion

The experience and training level of the three observers appeared to be an important factor affecting the identification of the end, neutral, stable vertebra, and FCRV, with a trend towards improved reliability of vertebral level assessment with increased experience, which is partly consistent with the finding by Benjamin et al. (9). The inherent difficulties in radiographic landmark identification and human error attributable to the “level ambiguity” that occurs when two (or even three) vertebrae nearly, could result in the discrepancies in the observer-selected levels (9). The UEV is defined as the most tilted vertebrae at the cephalad end of the main curve, human error attributable to the “level ambiguity” that occurs when two vertebrae nearly, but imperfectly, meet the criteria for the UEV could not be completely avoided (10). The NV is defined as the most cephalad vertebrae with apparently neutral rotation as assessed by pedicle symmetry within the radiographic silhouette of the corresponding vertebrae, human error in the “vertebrae rotation identification” is unavoidable when adjacent vertebrae are extremely close in rotation (9). The SV is the most cephalad vertebra caudal to the end vertebra of the main curve that is most nearly bisected by the CSVL, but there maybe a certain error in drawing the line erected vertically from the midpoint of S1, especially when the sacral anatomy is illegible (11). The FCRV is defined as the first vertebrae that presents opposite orientation of asymmetric HU ratio from the other vertebrae within major curve. For each measurement, the largest possible elliptical region of interest was drawn, but excluding the cortical margins to prevent volume averaging is a technically demanding manipulation, which may result in certain impact on measurement results. The accurate identification of radiographic landmark for the above vertebra is the embodiment of experience.

This is the first study to assess and compare the reproducibility and reliability of UEV, NV, SV, and FCRV interpretation in DLS patients, the intraobserver reliability was excellent for determining the FCRV, fair to good for determining the UEV, NV, and SV. FCRV is superior to UEV, NV, and SV in the accuracy of interpretation, two possible reasons may account for the difference. Firstly, in the identification of FCRV from CT examination, the measurement was performed within the concave and convex sides separately at three different regions of the vertebrae on coronal plane: immediately posterior to the anterior vertebrae margin, in the middle of the vertebral body, and anterior to the posterior vertebrae margin (7). Although excluding the cortical margins to prevent volume averaging is a technically demanding manipulation, it seems that the repeated measurement from different regions of the vertebrae could minimize measurement error when compared to UEV, NV, SV, which were measured only once from x-ray, while human errors attributable to the “level ambiguity”, “vertebrae rotation identification” should not be underestimated. Secondly, from a methodological point of view, the largest possible elliptical region of interest was drawn in the determination of FCRV, which is simple and rarely interfered by other factors (11–14). Contrarily, vertebrae rotation, osteoporosis, aortic calcification, and osteophyte hyperplasia, which may increase measurement difficulty and error in radiographic landmark identification, and would affect the accuracy of identification of UEV, NV, SV (15–17).

Benjamin et al. (9) demonstrated good to excellent intraobserver reliability in the radiographic determination of the EV, NV, and SV in adolescent idiopathic scoliosis (AIS) patients, which present more satisfactory results than the findings in the current study. The characteristics of the two different scoliosis render that there are bound to be differences in the identification of the vertebrae mentioned above. DLS is typically diagnosed in patients older than 40 years and without a history of AIS, the curves typically have an L2–3 apex and are associated with lateral olisthesis, rotatory subluxation, and structural vertebral deformity, which inevitably increase the difficulty in the identification of the EV, NV, and SV (18–20). Osteoporosis is also an important feature of DLS that distinguishing from AIS, and may obscure the radiographic landmark identification of the upper endplate and the pedicles of the vertebrae, finally lead to the variability in the identification of UEV, NV, SV that measured on x-ray (21, 22).

FCRV provides a meaningful reference in the selection of UIV for DLS patients, UIV above FCRV is superior to SV in reducing the incidence of proximal adjacent segment degeneration for DLS patients that received posterior fusion surgery (7, 8). The strength of the current study is that we firstly demonstrate FCRV is superior to UEV, NV, and SV in the accuracy of interpretation. Moreover, type C coronal malalignment, defined as CBD > 3 cm and a C7PL shifted to the convex side of the curve, is detected to be an important factor affecting the accuracy of FCRV identification. The alignment of the vertebrae proximal to the apex present smooth or flat in patients with Coronal imbalance type C, the difference of HU values between the convex and concave sides of these vertebrae is little, which may inevitably lead to a certain error in the identification of FCRV.

There are several limitations in the current study. First, the number of enrolled patients is relatively small, and from a single center, the number of Coronal malalignment type B and type C is relatively smaller when compared to type A, more DLS cases need to be included to verify this conclusion in the next study. Second, the subjects selected are all Chinese Han individuals, whether the conclusion is applicable to other ethnic groups needs to be further investigated in the future.

## Conclusion

Experience and training level of the observers are important factors affecting the accurate identification of these vertebrae in DLS, intraobserver reliability increases along with increasing levels of observer experience. FCRV is superior to UEV, NV, and SV in the accuracy of identification, Type C coronal malalignment could affect the accurate identification of FCRV.

## Data Availability

The raw data supporting the conclusions of this article will be made available by the authors, without undue reservation.
